# Considerations for the Use of Human Participants in Vector Biology Research: A Tool for Investigators and Regulators

**DOI:** 10.1089/vbz.2014.1628

**Published:** 2015-02-01

**Authors:** Nicole L. Achee, Laura Youngblood, Michael J. Bangs, James V. Lavery, Stephanie James

**Affiliations:** ^1^ Department of Biological Sciences, Eck Institute for Global Health, University of Notre Dame, Notre Dame, Indiana.; ^2^ Centers for Disease Control and Prevention, Atlanta, Georgia.; ^3^ Public Health and Malaria Control, International SOS, Papua, Indonesia.; ^4^ Centre for Ethical, Social and Cultural Risk, Li Ka Shing Knowledge Institute of St. Michael's Hospital, Toronto, Canada.; ^5^ Dalla Lana School of Public Health and Joint Centre for Bioethics, University of Toronto, Ontario, Canada.; ^6^ Foundation for the National Institutes of Health, Bethesda, Maryland.

**Keywords:** Vector, Entomology, Risk assessment, Epidemiology, Vector control

## Abstract

A thorough search of the existing literature has revealed that there are currently no published recommendations or guidelines for the interpretation of US regulations on the use of human participants in vector biology research (VBR). An informal survey of vector biologists has indicated that issues related to human participation in vector research have been largely debated by academic, national, and local Institutional Review Boards (IRBs) in the countries where the research is being conducted, and that interpretations and subsequent requirements made by these IRBs have varied widely. This document is intended to provide investigators and corresponding scientific and ethical review committee members an introduction to VBR methods involving human participation and the legal and ethical framework in which such studies are conducted with a focus on US Federal Regulations. It is also intended to provide a common perspective for guiding researchers, IRB members, and other interested parties (*i.e*., public health officials conducting routine entomological surveillance) in the interpretation of human subjects regulations pertaining to VBR.

## Introduction

Many diseases of public health importance, both within the United States and internationally, are transmitted to humans by an arthropod vector. In this review, vector refers to insects (mosquitoes, sand flies, tsetse flies), or acarines, (mites and ticks). Of greatest public health importance are mosquitoes and ticks that transmit diseases such as malaria, dengue, filariasis, hemorrhagic fevers, and viral encephalitides. Research to understand the vector as well as its interaction with humans and the environment (vector biology research [VBR]) aims to identify better ways to prevent the spread of such diseases and is a widely recognized field of biomedical research.

A thorough search of the existing literature has revealed that there are currently no published recommendations or guidelines for the interpretation of US regulations on the use of human participants in VBR. An informal survey of vector biologists has indicated that issues related to human use in vector research have been largely debated by academic, national, and local Institutional Review Boards (IRBs) in the countries where the research is being conducted and that interpretations and subsequent requirements made by these IRBs have varied widely. This review is intended to provide investigators and corresponding scientific and ethical review committee members an introduction to VBR methods involving human participation and to the legal and ethical framework within which these studies are conducted, with a focus on US Federal regulations. It is also intended to provide a common perspective for guiding researchers, IRB members, and other interested parties (*i.e*., public health officials conducting routine entomological surveillance) in the interpretation of ‘human subjects’ regulations pertaining to VBR.

**FIG. 1.**
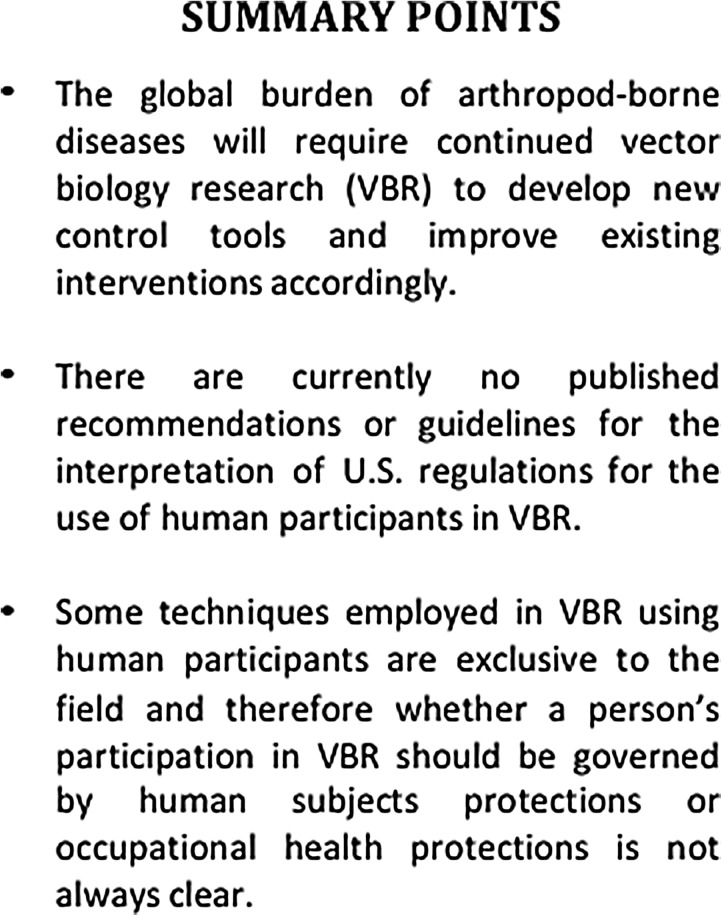
Summary points.

The specific aims of this review (Fig. 1) are to:

1. Describe the role of humans in common entomological techniques used in VBR;
2. Consider how VBR practices can be interpreted within the US regulatory framework;
3. Explain how these techniques are used to benefit science and public health and;
4. Develop a “living document” framework for which additional considerations can be easily accommodated and integrated.

The concept of a “living document” refers to the use of the current document structure as a framework to which additional considerations can be appended periodically to address an increasing number of specific VBR techniques. The current document focuses on the human-landing catch (HLC) technique, a method commonly used in routine surveillance (monitoring) and intervention evaluations for mosquito vectors. This comprises the first of a more comprehensive set of considerations related to additional VBR techniques (*i.e*., release of vectors in the field, human blood feeds for colony production).

Those using the information and references provided herein are advised that these considerations are only presented as guidance to assist in understanding VBR and not as mandates. They do not represent official policy of the National Institutes of Health (NIH) nor are they US Federal Regulations. We encourage those using them to be receptive to reasonable case-specific variations in practice that remain consistent with applicable policies on occupational, biohazard, and “human subjects” health and safety. These variations can be developed and evaluated in consultation with those proposing the research and external consultants who have expertise in the field.

## The Magnitude of Vector-Borne Diseases

Arthropod-borne diseases are responsible for an estimated 17% of the entire global burden of infectious disease (Townson et al. 2005), and as much as 50% of the world's population may be infected with an arthropod-borne pathogen (Institute of Medicine 2008). Moreover, in developing countries, a long-term trend of an increase in numbers of both cases and geographical distribution of malaria, leishmaniasis, bartonellosis, filariasis, dengue fever, and other arthropod-borne diseases has been observed (Gubler 1998). In some cases, these diseases are expanding due to vector adaptations, resistance to insecticides, and changing environmental conditions that are facilitating the creation of vector habitats and influencing human activities conducive to these diseases.

Malaria, which is caused by parasitic protozoa of the genus *Plasmodium* and spread by the bite of *Anopheles* mosquitoes, alone accounts for approximately 243 million clinical episodes of disease and 627,000 reported deaths annually (World Health Organization 2013a). The World Health Organization (WHO) review of the overall global malaria burden indicates that since 2000, mortality from malaria has fallen by more than 42% in all age groups and by 48% in children under 5 years of age (World Health Organization 2013a). In contrast, Murray et al. (2012) argue that mortality rates reflect significant misclassification and underreporting of malaria deaths, concluding that the actual number of deaths is close to 1.2 million, double WHO estimates. In either case, the global burden of disease from malaria remains unacceptably high.

Front-line malaria control interventions, including the use of long-lasting insecticide-treated nets (LLINs) and indoor residual spray (IRS) of insecticides, are among the most widely used and effective methods of arthropod-borne disease prevention. They have been shown to reduce disease burden dramatically when implemented properly (Roberts et al. 2000, Mabaso et al. 2004, Townson et al. 2005, Bhattarai et al. 2007, Fegan et al. 2007, Sharp et al. 2007, Chizema-Kawesha et al. 2010). Both act to kill the mosquitoes, shorten their life span, or otherwise modify their normal host-seeking behavior, thus reducing transmission (*i.e*., infective bites) to humans (Macdonald 1957, Grieco et al. 2007, Enayati and Hemingway 2010).

Despite past successes and continued global efforts to reduce the global burden of malaria and other vector-borne diseases, the utility of LLINs and IRS is threatened by the emergence of both insecticide resistance and behavioral adaptation in disease vectors. There is also growing public concern over the unintended consequences of pesticide exposure to humans, other nontarget organisms, and the environment. It is widely accepted that there is no “one-size-fits-all” strategy that can be universally successful across all disease ecologies (Enayati and Hemingway 2010). With the expanding global distribution of arboviruses (Weaver and Reisen 2010) and renewed calls for the elimination and eradication of malaria, it is inevitable that new tools and innovative strategies will be required to improve the impact of vector control programs accordingly (Alonso et al. 2010, World Health Organization 2010).

## The Role of Humans in VBR

### Vector biology research

The fundamental goal of VBR is to develop effective interventions to reduce the transmission of pathogens from vectors to humans by reducing the size of vector populations, or through specific mechanisms that interfere with the normal transmission capacities or behaviors of the vectors. Successful interventions depend on the prevention of human–vector contact to reduce or eliminate the probability of infectious biting—the predominant mode of pathogen transmission. This can be achieved through various strategies—reducing populations through the use of insecticides, biocontrol agents, or transfer of lethal genes; repelling and creating barriers against arthropod vectors; replacement of competent vector populations by refractory mosquitoes (*e.g*., infected with *Wolbachia*); trapping vectors from indoor and/or outdoor environments; as well as environmental modification or elimination of insect development sites. Each approach requires a thorough understanding of the basic ecology and behavior of the target vector for proper implementation and maximum impact for public health benefits. This includes preferred blood meal source, times of peak biting, location of biting (indoors or out), seasonal fluctuations in population densities, and characteristics that make particular habitats (development sites) preferred for successful vector production.

The ultimate objective of any vector control intervention is a reduction in human contact with infective arthropods. Quantifying this end point, before and after intervention, in longitudinal epidemiological studies involves: (1) Identifying and quantifying vector species and measuring vector population densities landing or biting on individual humans versus other animal hosts; (2) assessing shifts in vector population dynamics over time; (3) analyzing vector blood meals to determine host feeding preference(s); (4) assessing the abundance of vectors infected and infective with the pathogen; (5) estimating vector age; and (6) identifying and quantifying the vector species and population densities landing on humans indoors vs. outdoors. The standard tool for measuring critical entomological indicators of intervention effectiveness is assessment of direct human–vector interaction. The natural biological attraction of mosquitoes to humans is a complex, multivariate phenomenon and difficult to simulate. This information cannot be reasonably or accurately obtained in any other way that does not involve human–vector interaction.

Some techniques employed in VBR to generate information about human–vector interactions are exclusive to the field and therefore have unique characteristics that are challenging to consider in the framework of guidelines that were developed for more traditional areas of biomedical research (Council for International Organizations of Medical Sciences 2002, World Health Organization 2008). These unique VBR techniques include but are not limited to HLCs, vector trapping, blood feeding for vector colonization (or maintenance), infectivity studies, vector releases, and intervention trials. These methods can generally be divided into: (1) activities that relate to occupational safety; (2) activities that might have an indirect impact (*i.e*., epidemiological effect due to increased risk of pathogen transmission) upon the communities involved; and (3) activities that involve intervention or interaction with human participants, which may or may not be regulated for “human subjects” research. The last are regulated under Title 45 of the US Code of Federal Regulations, Part 46, Subpart A, known as the “Common Rule” which applies to research funded by the US federal government, whether it is carried out in United States or foreign institutions (US Department of Health and Human Services 2005). The Office for Human Research Protections (OHRP) compiles a comprehensive list of other national and international guidelines and regulations from around the world (www.hhs.gov/ohrp/index.html). These do not include any guidelines specific to VBR involving humans, although unpublished guides may exist for specific ethical committees. Distinguishing among the three broad VBR activity categories listed above and applying the appropriate standards can pose a challenge to reviewers, regulatory bodies, and ethicists in determining whether risks associated with the research have been minimized, and are reasonable in relation to the anticipated benefits.

### Examples of human participation in VBR

To increase familiarity with the methods of VBR, we highlight several examples of the types of activities that require human involvement or participation.

#### Attracting and collecting vectors

##### Experimental hut studies

**FIG. 2.**
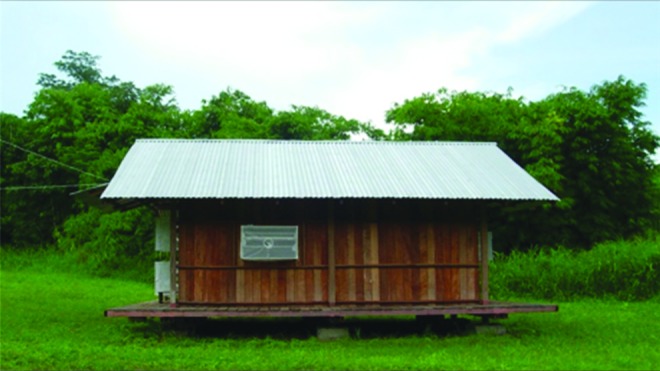
Experimental hut constructed specifically to evaluate vector bionomics. (Photo courtesy of Theeraphap Chareonviriyaphap, Katsetsart University, Bangkok, Thailand.)

Experimental hut studies are common tests used to validate laboratory findings of vector behavioral responses to insecticides (time and density of house entry or exit), perform small-scale field trials of interventions (IRS, LLINs), and describe natural ecology patterns of disease vectors (*e.g*., host choice; indoor vs. outdoor biting; resting preferences) both before and after treatment (World Health Organizaton 2006, 2009a, 2009b, 2013b, 2013c). There are two common types of experimental hut—newly constructed to serve specific study objective(s) (Fig. 2), and local housing that has already existed in the study area. In such studies, it is necessary to attract host-seeking vectors to the hut and collect them to assess the outcome of interest (*e.g*., a reduction in vector density in a treated hut compared to a control without intervention). Currently, there are no equally effective or near-equivalent substitutes for humans in terms of the public health relevance of understanding the response of vectors to humans in normal human habitations. In addition, when using interception traps on the entry and exit portals of experimental huts (e.g. windows or doors), captured insects must be removed throughout the study period by human collectors to accurately define temporal (*e.g*., hourly) outcome measures of repellency, irritancy, blood-feeding, and mosquito mortality effects of an intervention applied at the house level. There is also a requirement to observe vectors for indications of before-death (moribund) behaviors, such as the inability to fly or right oneself, which may be important outcome measures of insecticidal effects of the study interventions. At the current time, there are no effective alternatives to human collectors for performing these measurements.

##### Monitoring and surveillance

The sustainability of an intervention relies, in part, on the ability to measure its effectiveness over space and time through ongoing monitoring of vector densities and rates of infected specimens (James et al. 2014). These measures are gathered by human operators using standardized monitoring and surveillance techniques, including backpack aspiration, sweep netting, passive (ovitraps, resting boxes, etc.) and active (CDC light trap with or without additional attractants, etc.) trapping devices, as well as hand-held mouth, and/or mechanical, battery-operated aspiration devices to capture mosquitoes. These collection activities are carried out both inside habitations and outdoors. Another common indoor collection method is the pyrethrum spray catch that targets resting mosquitoes using a low-concentration insecticide as a knockdown agent. All of these methods require humans to manipulate the collection devices or implement the procedures.

#### Feeding vectors

Human blood for use in VBR can either be drawn via venipuncture from the human or vectors can be allowed to feed directly, for example, by exposing an arm of a human volunteer to a container of mosquitoes allowing them to take a blood meal. One purpose of allowing vector populations to take blood meals from humans is to establish working colonies of the target insect in a laboratory setting. This allows researchers access to vector populations as needed to investigate various biological, ecological, and/or pathogen characteristics important to modeling transmission dynamics under controlled conditions. It is common that vector populations collected from the field (*i.e*., wild-caught) do not feed readily on nonhuman animal blood during early generations. This makes the establishment of colonies extremely challenging without reliable access to human blood in some form. In addition, although artificial membrane feeding systems exist, in which mosquitoes are required to land on and bite through a membrane surface of a container of human blood, wild-caught vectors typically require a period of “training” before readily accepting blood under these conditions. The primary reasons for using humans rather than an alternative animal blood source include: (1) anthropophagy (some mosquitoes will only feed on human blood); and (2) differences between human and animal blood (proteins and their constituent amino acids) that could affect vector fitness (*i.e*., survival) and therefore behavior (Prasad 1987).

Another purpose for human feeds is to investigate host–vector–pathogen relationships. To study natural vector–pathogen cycles, the most appropriate method is to identify naturally infected humans from areas with endemic transmission and allow uninfected arthropods to feed on blood from those likely to be infected and then to track pathogen development within the vector. Recruitment for such evaluations typically occurs at local health clinics where persons present with signs and symptoms of the target diseases that can be properly diagnosed and subsequently treated, if feasible (*i.e*., arboviral infections vs. malaria infections). Such feeds are important to define and understand the pathogen development cycle (*i.e*., “competence”) within the vector (developmental stages, susceptibility, tissues infected) so that intervention targets can be identified to interrupt transmission (*e.g*., develop transmission blocking vaccines, novel chemicals, genes involved in development) (Klein et al. 1991, Sahu 1998, Vaughan et al. 2007). Similarly, human feeds are also used in vaccine challenge studies, in which experimental vaccine recipients are exposed to laboratory-infected mosquitoes to test the ability of the vaccine to prevent infections (commonly used in malaria vaccine development) (Seder et al. 2013, Kamau et al. 2014) or to determine what virus titers (*e.g*., dengue) are necessary for human infection (Lyons 2014).

Although animal models of pathogen transmission, including those in nonhuman primates, can provide valuable information, the models often do not predict important aspects of disease progression and outcomes in humans. For example, components in vector saliva (bioactive molecules) are known to affect the success of pathogen infection and outcomes of disease in humans (Leitner et al. 2011). Observation of the interaction of salivary compounds from specific insect vectors with the human immune response, in association with the pathogen of interest, is necessary to predict human responses more accurately because these may be different from the response in primates. Similarly, the efficacy and safety of antipathogen drugs or vaccines must be tested in humans at some point in the development process and the information gathered from evaluating host–vector–pathogen relationships using human feeds can be integrated into these models for greater predictive accuracy.

#### Releasing vectors

Research that requires the release of mosquitoes falls primarily into two categories: (1) studies to improve understanding of mosquito behavior and ecology and (2) studies to control/manipulate medically important mosquito populations. Typically, adult insects are marked with fine fluorescent powder prior to release to track movement patterns and flight distances and to estimate survival rates over time (Lacroix et al. 2009). Vector populations used in these studies can originate from either captured adult insects that are collected on site or from immature forms also collected at site that are reared to adults. Vector populations can also originate from laboratory colonies that have undergone manipulation (*e.g*., irradiation) prior to release. Experiments with field populations are required to test hypotheses resulting from theoretical models and laboratory studies. Although elegant cages simulating natural field conditions have been developed and can provide a useful tool as an intermediary bridge between laboratory and field conditions, they remain a simulation of the environment nonetheless (Ng'habi et al. 2010). Furthermore, the end goal of multiple VBR research projects, including vector population suppression and population replacement projects, ultimately requires humans to release large numbers of mosquitoes into the field.

## The Ethics and Regulation of Human Participation in VBR from the Perspective of US Federal Regulations

### Key criteria defining human subject research

In the United States, the primary regulation governing the protection of human research subjects is the Common Rule (45 CFR 46, Subpart A) (US Department of Health and Human Services 2009). Because much of VBR is conducted outside of the jurisdiction of the United States and its territories, it is important to delineate the scope of this policy. As specified in the Common Rule, “…this policy applies to all research involving ‘human subjects’ conducted, supported or otherwise subject to regulation by any federal department or agency….” Additionally, “It also includes research conducted, supported, or otherwise subject to regulation by the federal government outside the United States.” [45 CFR 46.101(a)] (US Department of Health and Human Services 2009). Thus, the policy applies to research funded by the US departments and agencies that have adopted the Common Rule, including the Department of Health and Human Services (HHS) and the Department of Defense (DoD), even if the research takes place in another country. Compliance with the regulations is necessary to be eligible to receive US federal funding for the research.

Research supported by non–US government entities and not using US government resources is not subject to the policy unless US government employees or agents participate in the conduct of the research, or unless participating institutions have a Federalwide Assurance (FWA) with the OHRP that stipulates its intention to follow the Common Rule regulations in all research conducted under its auspices. Research under the purview of this policy must also comply with the respective national policies and judgments of local IRBs in place within the country where it is being conducted. It is incumbent upon researchers and their institutions to seek out, understand and ensure compliance with the national policies of the country where research is being performed.

**FIG. 3.**
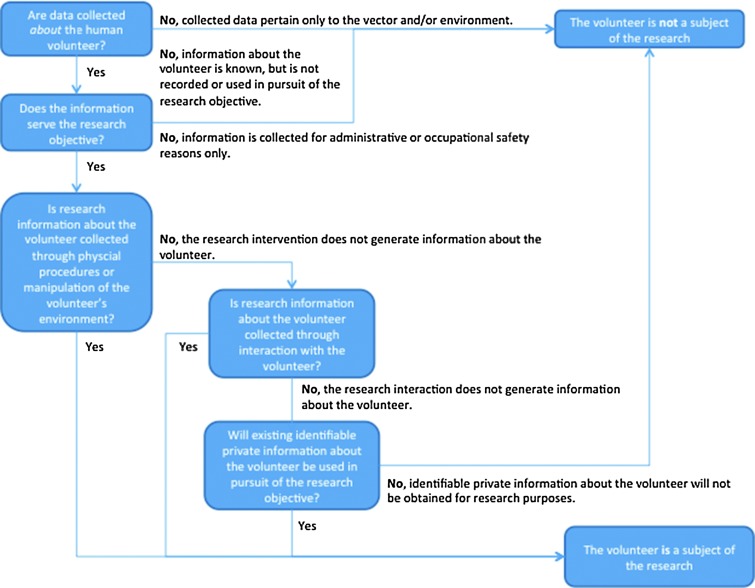
Decision points regarding the definition of a “human subject” in vector biology research (VBR).

Whether or not an activity is considered “human subjects research” according to the US regulations, and is therefore subject to its provisions, is determined by several key tests, outlined in the regulation (www.hhs.gov/ohrp/policy/ohrpregulations.pdf) and discussed below. A series of decision points regarding the definition of “research” and “human subjects” pertaining to this discussion can be found in Figure 3 and Appendix I (Supplementary Data are available at www.liebertonline/vbz).

#### Is the activity research?

As defined in US regulations, “research” means a systematic investigation, including research development, testing, and evaluation, designed to develop or contribute to generalizable knowledge. Activities that meet this definition constitute research for purposes of the Common Rule regulations, whether or not they are conducted or supported under a program that is considered research for other purposes. For example, some demonstration and service programs may include research activities [45 CFR 46.102(d)] (US Department of Health and Human Services 2009). “Generalizable knowledge,” while not formally defined, is often considered to refer to new knowledge that furthers the scientific or academic understanding of a particular topic of study or discipline.

Some activities, such as providing blood for the continuous maintenance of vector colonies, do not contribute directly to generalizable knowledge and can be classified as occupational activities whose hazards (minimal) have been addressed by existing guidelines, *e.g*., Occupational Safety and Health Administration (www.osha.gov) Blood-borne Pathogens Standard [29 CFR 1910.1030]. If the activities are performed in support of a specific research objective(s), they are classified as research. However, the research may not necessarily involve “human subjects” as defined by the regulations.

Although the focus of this review is on VBR methods, the authors wish to acknowledge that these same methods may be employed outside the context of research, in which case they may not be subject to the regulatory requirements for research.

#### Does the research involve “human subjects”?

A “human subject” is a living individual about whom an investigator (whether professional or student) conducting research obtains: (1) data through intervention or interaction with the individual or (2) identifiable private information. “Intervention” includes both physical procedures by which data are gathered (*e.g*., venipuncture) and manipulations of the subject or the subject's environment that are performed for research purposes (see What is an intervention in VBR?, below). “Interaction” includes communication or interpersonal contact between investigator and subject (see What is an interaction in VBR?, below). “Private information” includes information about behavior that occurs in a context in which an individual can reasonably expect that no observation or recording is taking place, and information that has been provided for specific purposes by an individual and that the individual can reasonably expect will not be made public (*e.g*., a medical record). Private information must be individually identifiable (*i.e*., the identity of the subject is or may readily be ascertained by the investigator or associated with the information) for obtaining the information to constitute research involving “human subjects” [45 CFR 46.102(f)] (US Department of Health and Human Services 2009).

**If a researcher obtains data about an individual** ***for research purposes*** **through interaction or intervention with that individual, then that individual is a human subject,** ***whether or not the information is identifiable or private*.**

**Example:** An anonymous survey conducted for research purposes, in which respondents are asked questions about themselves would be considered research involving “human subjects” because information is being collected about living individuals by interacting with them.

**Conversely, if identifiable private information about a living individual is obtained** ***for research purposes*, then that individual is a human subject,** ***whether or not the information was obtained through interaction or intervention with that individual*.**

**Example:** Use of an existing database that includes identifiable private information about living individuals to perform a research analysis is still research involving “human subjects”, even though the researcher has not obtained the data through direct interaction or intervention with those individuals.

**Data collection obtained through interaction or intervention with a living individual is considered “human subjects” only** ***if the data are about the individual*** **with whom the researcher interacts or intervenes.**

**Example:** A research survey in which questions are limited to information about the facility where the respondent works is not research involving “human subjects” because information is not being obtained specifically about that individual.

However, judging whether information being collected is about the individual, or ruling out that the information is *not* about the individual, may not always be straightforward and in many cases will require reasoned judgments.

##### What is an intervention in VBR?

As defined above, intervention includes both physical procedures and manipulations of an individual's environment through which data are gathered about that individual. It is important to note that an intervention, on its own, does not necessarily categorize an individual as a subject of research. It is the collection of research data about the individual that causes one to be a subject of the research.

**Example 1:** A volunteer donates blood to feed a colony of mosquitoes that is being maintained for a specific research activity. No information about the volunteer is recorded.

Although blood donation is a physical procedure being performed for a research purpose, no data about the volunteer are being obtained. Therefore, the volunteer is not a subject of the research in this example. However, if the blood were tested to assess the impact of human factors (*e.g*., blood type, metabolic factors, pregnancy) on mosquito feeding preferences, then the volunteer would be a subject of the research, because data about the volunteer are being obtained through the physical procedure being performed for research purposes.

**Example 2:** An individual who was naturally infected with a pathogen of interest and diagnosed in the course of regular clinical care volunteers to allow uninfected vectors to take a blood meal from an exposed limb before receiving clinical treatment. No information about the volunteer is recorded.

Although this is a physical procedure being performed for a research purpose, no data about the volunteer are being obtained, therefore the volunteer is not a subject of the research. In addition, although the volunteer's infection status is personal information, it was initially obtained for clinical purposes, and it is not recorded for purposes of the research. Therefore, the volunteer is not a subject of the research in this example. However, if information about the infected person (*e.g*., age, gender, human immunodeficiency virus [HIV] status) is collected as part of the data required to answer a research question, such as the impact of various human host characteristics on transmission of pathogens to mosquitoes, then the volunteer would be a subject of the research, as data about the volunteer are being obtained through the physical procedure (intervention) being performed for research purposes.

**Example 3:** A researcher is using HLCs to evaluate an intervention designed to reduce mosquito density in a malaria-endemic community. A project staff volunteer is tested for malaria after the research is completed and/or their participation with the project stops to provide treatment in case of infection (see “Human Subjects”/IRB vs. occupational health/biohazard, below).

In this example, the testing is not done for the purpose of answering a research question, but for the occupational safety of the volunteer. Although mosquitoes are ideally collected before they bite, there is always the possibility that the collector did not capture a mosquito before the biting sequence begins (*i.e*., probing and injection of salivary gland secretions). The volunteer will be provided treatment if the test is positive. Thus, the information about the volunteer was not obtained to serve a research objective and as such the research would not be considered to involve “human subjects.” Additional protections are provided to the volunteer as occupational safety measures, not as human research protections.

##### What is an interaction in VBR?

“Interaction,” as described in the Common Rule, refers to communication or other interpersonal contact between a researcher and an individual for the purpose of collecting personal data about the individual for research purposes. Interaction that does not result in the collection of information, such as providing instructions to a volunteer on how to collect mosquitoes, does not define an individual as a research subject. Interaction that does not serve the research objective, such as collecting a volunteer's contact information to facilitate compensatory payment for his time, does not define an individual as a research subject.

### Obtaining identifiable private information

Research using information that is not obtained directly through intervention or interaction (*i.e*., secondary use of data collected for other purposes) may fall within the scope of “human subjects” research regulations, if the data contain, or are linked to, identifiable private information. These types of research activities are uncommon in VBR, and where they are applied, the ethical considerations and regulatory interpretations are not unique to this field.

### “Human subjects”/IRB vs. occupational health/biohazard? (see Appendix I)

Like all research activities in which infectious agents are involved, vector biology studies may entail the occupational risk of acquiring infection in both laboratory and field research settings. Laboratory-acquired infections have resulted from exposure to vector-borne agents including both arboviruses (American Committee on Arthropod-borne Viruses 1980) and protozoan parasites (Herwaldt 2001). Measures for mitigating these risks have been developed and widely implemented. These can consist of strict containment and manipulation measures, worker training, and working with, where appropriate and feasible, related organisms that are not pathogenic in humans (e.g., *Plasmodium berghei*, a rodent malaria).

Whether a person's participation in VBR should be governed by “human subjects” protections or occupational health protections is not always clear, because the underlying goals may be the same, but the rationales and criteria for triggering each set of protections may be very different. Most VBR programs employ individuals to carry out some of the activities, described above, as full- or part-time staff members, as opposed to episodic engagements. The hiring process includes outlining primary responsibilities that may include interaction with vectors such as colony maintenance, HLCs in natural or semi-field conditions and/or vector releases to name a few examples. These participants are then expected to follow standard laboratory practices as outlined by the project leader and specific research institution's specifications to protect them from occupational hazards and biohazard exposures. However, when information about the employee is obtained or used in pursuit of a research objective the employee becomes a research subject and “human subjects” research regulations would apply, in addition to any applicable occupational health or biosafety requirements.

Human participation in VBR and the relevant activities outlined in this document may fall under either “occupational hazard” or “human subjects” categories, or both. Similarly, paid project staff may fall under occupational safety guidelines or “human subjects” guidelines, or both. There is no doubt some subtleties exist that can create confusion. Even if there is some inherent risk involved with participating in a research project, the individual may not be considered a human subject. However, such individuals remain entitled to protection of their rights and welfare and to the general respectful treatment that would otherwise be required if defined as “human subjects.”

## Techniques in VBR Involving Humans

### Technique I—human-landing catches

#### What are HLCs and why are HLCs performed?

HLCs have been used for decades and are considered the most effective method for collecting mosquito species to assess the frequency or nature of vector–human contact (World Health Organization 1975, Silver 2008). HLCs are a standard tool in VBR because there is no known stronger attractant for anthropophilic mosquitoes than humans (combinations of movement, body heat, “odorants,” and other chemical emanations) and thus HLCs represent the most direct, accurate, and reliable method of estimating the true exposure of humans to vectors (biting pressure).

**FIG. 4.**
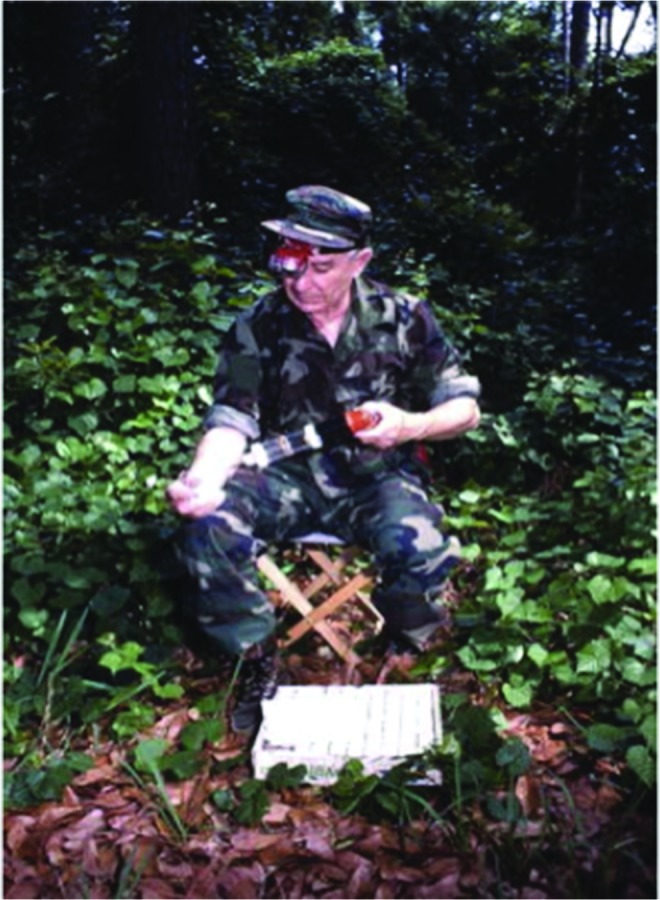
Human-landing catch (HLC) technique in an outdoor setting. Mosquitoes landing on the exposed extremity (arm or leg) are collected using a battery-operated or mouth aspirator. (Photo courtesy of Carl Schreck, USDA-AR, Gainesville, FL.)

The basic technique involves exposing arms or legs of human collectors. Host-seeking arthropods, (typically mosquitoes and flies) that land on the skin surface are captured with manual (mouth) or mechanical (battery-operated) aspirators before biting and then transferred into suitable holding containers (Fig. 4) (World Health Organization 1975, Silver 2008). HLCs can be performed in a variety of settings, such as inside houses, animal shelters, and various outdoor locations, including inside village compounds.

The HLC method is used to describe the general ecology of target species—seasonal distribution, peak biting times, and indoor and outdoor biting preferences. HLCs are also used to estimate flight distances, evaluate the efficacy of transmission control tools, such as topical repellents, bed nets, and chemical insecticides, as well as to describe vector species compositions in a particular study site. In field studies, the vectors can be further analyzed for infection rates to estimate pathogen inoculation (transmission) rates, identify and incriminate primary, secondary, and incidental vector species, determine pathogen species composition, and compare before and after intervention landing rates to monitor effectiveness of a control strategy. HLCs are employed under laboratory, semi-field, and field conditions based on research goals and therefore can vary in the risk of potential adverse effects to humans. HLCs have been most widely used for monitoring the human-biting mosquito population that is epidemiologically relevant to transmission and control of malaria, arguably the arthropod-borne disease with greatest global health burden (World Health Organization 2013a). Because HLCs provide a sensitive measurement of mosquito population presence and density, this method is expected to continue to be a vital tool in the coming years as national vector control programs shift their malaria agenda from control to elimination, which, depending on the design of the program, may suppress mosquito populations and make them more difficult to measure.

Without doubt, the majority of vector control strategies and tools used to prevent arthropod-borne disease today could not have been discovered, conceptualized, developed, and optimized without the use of HLCs. Improved understanding of adult vector species population composition and geographic distribution and the discovery of “new” arthropod-borne diseases can also be attributed to the use of HLCs in VBR. Despite inherent bias (*e.g*., overestimation) and random error associated with HLCs, no single or combined methods for adult mosquito collections and monitoring has yet proven capable of matching or replacing HLCs to estimate human–vector contact accurately and consistently. Therefore, HLCs remain a critical component to understanding the dynamics (time and space) of vector-borne disease transmission and remain the gold standard for determining human contact with vectors.

#### Applications and impact of using HLCs in VBR (see Appendix II)

##### Vector bionomics

Bionomic (ecology) studies routinely employ HLCs to identify those vectors that are more prone to biting humans and thus contribute to greater risk of pathogen transmission. Determining the intensity of parasite transmission by vector populations is a key component of epidemiological studies of arthropod-borne diseases. Two important mathematical approaches for estimating the risk of vector-borne disease transmission are the entomological inoculation rate (EIR) and vectorial capacity (VC). EIR is an estimated measure of the average number of infective bites each person receives per night (or other measure of unit time) and is a direct measure of the risk of human exposure to the bites of infective mosquitoes (Beier 2002). VC measures the potential for pathogen transmission (or “force of transmission”) on the basis of several key biological parameters of vector populations to include the probability that a mosquito will take a human blood meal (Macdonald 1957). The VC formula expresses the relative ability and efficiency of a vector population to transmit malaria on the basis of the potential number of secondary inoculations originating per day from an infective person (gametocyte carrier). The EIR is the only direct measure of malaria transmission and the only useful index for predicting malaria epidemics (Onari and Grab 1980). Both EIR and VC are essential components of virtually all mathematical models of malaria transmission (Macdonald 1957, Dietz 1974, Najera 1974, Pull and Grab 1974, Bailey 1982, Saul et al. 1990, Eckhoff 2011, Wallace et al. 2014), and therefore critical to have accurate vector biting attack rates. Human-landing rates used in both mathematical formulas are best estimated by performing HLCs throughout the range of expected biting period and can provide measures of probability of exposure by indoor versus outdoor location, time, and frequency of greatest exposure (*i.e*., hours of peak biting) and seasonal patterns of risk. Other methods fail to provide this, thus this is one substantial reason why HLC is performed and needed.

##### Vector control product development

Research and development for novel vector control products employ HLC techniques to assess effectiveness. Products evaluated using HLCs include those designed for personal application, such as the topical repellents (*e.g*., *N*,*N*-diethyl-meta-toluamide [DEET]) (McCabe et al. 1954), pyrethroid-treated clothing, or other insecticide-treated materials (ITMs), as well as those designed to be applied at the household and community level such as LLINs and active ingredients for use in IRS. Analytical-grade ingredients used in these interventions and the formulated product themselves are typically evaluated in a stepwise manner starting with laboratory studies that might include cage-trials with colony-reared mosquitoes (Phase I) then progressing either to semi-field settings in biospheres (Ferguson et al. 2008) using colony vector populations (Phase II) or to full-field conditions using experimental huts situated within natural vector populations (combination Phases II and III). Standard protocols for these study designs have been developed by the US Environmental Protection Agency (EPA) (US Environmental Protection Agency 1999) and the World Health Organization Pesticide Evaluation Scheme (WHOPES) (World Health Organization 2006, 2009a, 2009b, 2013b) and include the use of HLCs in varying forms.

There is currently no satisfactory test procedure to evaluate the insect bite protection performance of repellent-treated clothing or other repellent consumer products (*i.e*., coils, candles, etc.) without the use of humans. The use of a knockdown (KD) response (the inability of a vector to right itself following exposure to product) as the primary measure of efficacy is insufficient for products designed to prevent bites and not necessarily kill (WHO 2013b). HLC provides the most accurate “weak point” assessment of new products because there is no current stronger attractant for anthropophilic mosquitoes (those attracted to humans, especially as a source of food) than humans, and HLC measurement with participants who do not receive treatment in the experimental design controls for mosquitoes' preferences on the basis of variations in individual participant's attraction levels.

Observations of how vector control chemicals function to prevent arthropod-borne disease transmission were conducted under field conditions starting in the 1960s using human collectors inside experimental huts (Smith 1963, Zulueta and Cullen 1963, Smith and Webley 1969). Experimental hut structures are designed to mimic typical households but under more “controlled” settings in the field. They are the standard means of discovering novel active ingredients (synthetic or natural) that may have greater effectiveness than current commercially available products. Experimental hut studies are commonly used to validate laboratory findings of vector behavioral responses to insecticides, describe patterns of entry and exit behavior pre- and posttreatment, and serve as small-scale field trials of interventions (*e.g*., IRS, ITNs). In such studies, there is often a necessity to attract host-seeking vectors to the hut to measure efficacy, *i.e*., a reduction in vector density and/or blood feeding compared to a matched, untreated control hut. Efforts to identify and reproduce human host cues (lures) are ongoing, but currently there are no effective substitutes for the use of humans in generating comparable human host cues to attract mosquitoes when in direct comparison (Okumu et al. 2010) or from both long- and close-range distances (Bernier et al. 2007, Smallengange et al. 2010). Additionally, when using interception traps on the portals (windows, doors, eaves) of experimental huts, the captured insects must be removed and recorded periodically throughout the study period to accurately describe effects/action end points (*i.e*., excitation/repellency) by time interval (*e.g*., hourly). There is also a requirement to observe the potential KD effect of a chemical inside structures as an important mode of action to reduce potential biting and pathogen transmission. At the current time, each of these measurements cannot be performed without human collectors being directly involved due to the various mechanical and observational requirements of the study designs.

##### Technological advances

The improvement of advanced technologies has been driven by activities and findings from HLC. This includes helping to establish the molecular techniques used to accurately identify parasite infections in natural vector populations that routinely feed on humans, because human collectors are used to generate the critical samples required for laboratory analysis. Accurate species identification is vital to our understanding of vector pathogen transmission and applying effective vector control measures. Due to the problematic nature of accurately identifying species of many vectors within “complexes” on the basis of existing morphologic keys, molecular methods for confirming the true vector species status and for identifying ambiguous or damaged field samples have also benefited and advanced disease epidemiology through HLC studies. In addition, many “complexes” contain both anthropophagic (human feeder) and zoophagic (animal feeder) species, and HLC has served to distinguish this characteristic.

##### Control program development

Finally, the development of effective vector control programs would be highly compromised without the use of humans to collect adult vectors. The design and implementation of organized programs requires a thorough understanding of site-specific transmission dynamics to include all aspects of vector bionomics (species composition, vector status, infection rates and comparative susceptibility, seasonal densities, location of human/vector contact [indoor:outdoor biting ratios], response to environmental parameters, and insecticide resistance status among others). Such vector bionomic data is gathered through HLCs and the manual collection of vectors from inside structures (*e.g*., resting collections) or trapping devices—all of which require human participation. The information is used either directly “on the ground” to implement operational changes in control activities or indirectly in disease transmission modeling efforts to calculate EIRs and other useful entomological parameters to better understand and monitor program effectiveness and identify factors for possible exploitation. Both approaches can drive maximum cost effectiveness using currently available vector control tools and advance the development of improved programs for the future.

#### Possibilities for using alternate technology to replace humans

Because of the inherent risks of conducting HLCs in areas that are endemic for vector-borne diseases, many researchers have attempted to use other sampling devices and correlate their measures with HLCs, but no universally satisfactory or acceptable alternative to HLCs has been developed to date. Variations on the basic HLC model include enclosing humans acting as “bait” attractants in nets, cages, or traps that allow mosquitoes to enter, but not escape, while protecting the human from direct mosquito contact (Silver 2008). Even host-seeking trapping devices that are designed to replace the use of humans in VBR require the use of human collectors to compare effectiveness during development trials.

Methods of evaluation include the use of human collectors in wind-tunnel tests under laboratory conditions and the use of HLCs under field conditions to measure collection rates between humans and various other trapping methods. Many sampling methods have been evaluated as possible alternatives to HLC with varying degrees of success depending on mosquito species, study areas, research methodology, and analysis (Service 1977, Kilama et al. 2014, Lima et al. 2014). The issue becomes one of interpretation of trap data as an accurate reflection of the actual human-biting population (Husbands 1976). For the estimation of malaria transmission intensity, however, it is an important prerequisite that the sampling methods used are calibrated against the HLC (*i.e*., near equivalent to “biting” density per person/unit time) as the “gold” standard (Githeko et al. 1996). This is because the HLC translates directly into human biting rates, which serve as an essential parameter in the estimation of both the EIR and VC used in monitoring control program success (or failure) and in developing transmission models (Macdonald 1957, Garret-Jones 1964, Garret-Jones and Shidrawi 1969, Garret-Jones 1974, Dye 1986, Freier 1989, Saul et al. 1990, Mboera 2005, Wallace et al. 2014, Eckhoff 2011).

#### Ethical and legal decision factors for human participation in HLCs

The ethical considerations surrounding HLCs are complicated by issues, such as the potential of integrating vulnerable participants such as children (Lindsay et al. 1995), or people living in poverty who could be susceptible to undue inducement in the form of incentives for carrying out HLCs (Aultman et al. 2000, Kilama 2010), even though they might be exposed to these vectors under their routine living conditions. In fact, a recent study has indicated that HLC collectors provided appropriate malaria prophylaxis are at significantly lower risk of pathogen infection than noncollectors that are within the same study area (Gimnig et al. 2013).

However, some important vector-borne diseases, such as arboviruses and leishmaniasis, have no effective chemoprophylaxis, preventive vaccines, or effective treatment (*i.e*., dengue) for individuals who become infected. In these situations, the risks associated with acquiring an infection through HLCs are categorically different than those with diseases for which there is effective treatment. Providing adequate protection for HLC collectors under these scenarios, therefore, may require different strategies to minimize risk, whether the activities are deemed to be research or routine occupational exposures (Aultman et al. 2000, Andrade-Narvaez et al. 2009). These include alternative collection methods, such as having participants wear protective clothing to cover areas of the body not being used to collect vectors or using humans solely as an attractant source to draw vectors to a location without exposing any portion of the body to vector biting.

HLCs are typically conducted by study staff and/or local employees as part of their work responsibilities or by volunteers recruited by the community. In most research designs, these HLC collectors are not research subjects (see Does the research involve “human subjects”?, above). However, occupational health and safety standards require that sufficient measures must be taken to protect staff from unacceptable occupational hazards associated with HLCs in natural transmission settings, where the same measures taken for reducing risk of exposure to infection in the laboratory cannot be applied (*i.e*., arthropod containment) (see Risk mitigation practices to consider for HLC and Summary of ethical considerations for conducting HLCs, below). Researchers must also consider whether “human subjects” research regulations, *i.e*., the Common Rule, apply. As discussed above in the Regulations section, a “human subject” is any living individual about whom data are obtained through interaction or intervention. In the context of HLCs, collectors may meet the definition of “human subjects,” regardless of their employment status. Such HLCs are subject to the requirements of the Common Rule, including the requirement to obtain IRB review.

There are scenarios where human participants conducting HLCs may satisfy the definitions to be both research subjects and employees such that the information gathered from HLC collectors has some role in answering a research question and is not collected anonymously (see Appendix I). An example would be measuring true risk of pathogen infection as a direct result of HLC collections using infection incidence as the outcome. In this case, blood samples are provided by HLC collectors and compared to persons not conducting HLCs throughout a set time period. Under these circumstances, protections are required to meet both research subjects and occupational health and safety standards, and where there may be conflict in these protections, those that provide the greater protection should be used.

Regardless of the applicability of the Common Rule, researchers employing HLC methods must adhere to any locally applicable guidelines, as well as accepted principles that underlie ethical standards including those outlined in the Belmont Report—respect for persons, beneficence, and justice (Department of Health, Education, and Welfare 1978).

##### Risks and benefits

An important consideration in any research activity involving human participants is the assessment of risks and benefits. Risks to participants must be minimized to the fullest extent possible, and they must be reasonable in relation to the anticipated benefits to the participants or their communities, or in light of the importance of the knowledge to be gained from the research. Although these types of studies do not often hold out the prospect of direct benefit for participants, they do include the possibility of enhancing public health through the results of the research. Research risks of HLC, on the other hand, can include minor adverse events such as transient discomfort (which might be considered a minimal risk), irritation or even allergic responses due to mosquito bites, and, more seriously, contraction of a vector-borne disease, such as malaria, dengue, and other pathogens, which might present varying levels of hazard depending on the disease and circumstances. The risks are obviously higher as disease endemicity (intensity) and/or severity increases in study areas. In highly endemic areas, some researchers opt to exclude HLCs due to the high risks of collectors contracting an infection, especially in the absence of vaccines, effective prophylaxis, and/or treatments (Aultman et al. 2000, Kilama 2010).

A public health–related risk might arise if the collector has been infected with a mosquito-borne disease prior to the engagement in HLC activity, but the probability of mosquito transmission might be reasonably argued to be equal, or possibly greater, had the collector been in his/her own environment, and can be partly mitigated by testing the individual for vector-borne infections before commencing HLCs (Gimnig et al. 2013). A key question for IRBs in many cases may be the extent to which the outcomes of concern are attributable to the research.

##### Informed consent

Efforts should be exerted to protect participants' well-being and reinforce their autonomy. HLC collectors have the right to complete and accurate information on material risks in fiduciary relationships, or in relationships (*e.g*., employer–employee) when there is some recognized duty of care. In those cases in which HLC collectors are “human subjects,” specific regulatory requirements of informed consent in research must be followed (Council for International Organizations of Medical Sciences 2002, World Health Organization 2008). Participants should be told about all the materially relevant risks and informed what will and will not be provided in the event of a research-related injury. In the case of HLC and other VBR scenarios, it might be especially important to explain, in advance, what might constitute a research-related risk, *i.e*., in experimental hut studies, the research-related risk may be exactly the same risk that people experience in their day-to-day lives (*e.g*., exposure to high indoor temperatures, potential mosquito bites, etc.).

Even in the absence of a specific regulatory requirement, such as when HLC collectors are employees and not subjects of the research, there is an ethical obligation to ensure that participants enter into research voluntarily, on the basis of complete and accurate information (Department of Health, Education, and Welfare 1978). In the context of HLCs, collectors should be fully informed about the nature, purpose, procedures, risks, and benefits of the research and any protective measures that will be supplied, and that their participation and withdrawal are voluntary and nonpunitive and then consent to agree or not agree to become employees. Participants should also be advised of any rights or services that are available to them, such as free diagnosis and treatment should they contract an infection or develop adverse reactions to the bites or infection, if applicable (Belsky and Richardson 2004, Hooper 2010).

#### Risk mitigation practices to consider for HLCs

To mitigate or avoid the risks stemming from potential infective vector bites, it is recommended that whenever possible, HLC participants wear protective clothing (*i.e*., mesh jackets, closed-toe shoes) to cover areas of the body that are not being used to collect vectors and to minimize vector contact with multiple sites of exposed skin. Training of collectors should be conducted prior to performing HLCs to maximize the capture rate of landing vectors before they have had a chance to insert their mouthparts and probe/bite (Silver 2008). However, because this might not be possible in every instance, it is advisable that collectors take routine prophylactic medicines (*e.g*., antimalarials) or are vaccinated (*e.g*., for yellow fever) when these precautions are available, feasible and applicable (Council for International Organizations of Medical Sciences 2002, World Health Organization 2008). The taking of chemoprophylaxis or vaccination should remain voluntary provided the participant understands the possible consequences of not taking a preventive measure (*i.e*., risk of infection) and provides consent. However, depending on the circumstances, preventive measures could be mandatory for participation (*e.g*., vaccination required for collecting forest vectors in a yellow fever endemic area). Collectors should also be monitored periodically to detect subclinical infections. Investigators have an obligation to inform participants during the consent process as to what medical treatment services are available in case a vector-borne disease is contracted as a result of HLC participation and whether specific treatment (*i.e*., only as it pertains to the risks from HLC and not medical conditions unrelated to the collection activity) will be free-of-charge to participants.

### Summary of ethical considerations for conducting HLCs (Fig. 5)

1. HLCs should be used only when scientifically justified (*e.g*., no near-equivalent substitute trapping/collection method available).
2. Researchers should be aware of all known actively circulating vector-borne diseases prevalent in the study area and take steps to ensure safety and to manage exposure as best as possible without compromising the study objectives.
3. When recruiting participants for vector collection, researchers should pay special attention to vulnerable populations (*e.g*., children) and consider exclusion criteria.
4. Informed consent should always be sought from participants. If research does not fall under 45 CFR 46, (*i.e*., “human subjects” research), the consent process need not conform to the requirements of §46.116, but participants should still be educated regarding the terms of their participation, the risks to which they may be exposed, and the measures that will be taken to minimize those risks. For HLC participants, protective clothing should be provided so that the area of the skin used to attract the vector is as limited as possible and the area where the vectors are expected to land is clearly visible for quick identification of its presence and rapid collection before the insect has the opportunity to bite.
5. As practicable, researchers should consider screening the participant for endemic vector-borne diseases in the area of study using a blood test before their participation (note that in malaria-endemic areas where participants are likely to be parasitemic, screening for active disease and treatment during the course of the study may make better sense).
6. When studying diseases for which prophylactic drugs are available, researchers should consider providing such drugs to participants, taking into account the probability and magnitude of harm associated with the disease and with the prophylaxis, as well as the availability of safe and effective treatments. There may be cases where it would be better not to provide prophylaxis—or to allow participants to choose—such as if the prophylaxis itself has unacceptable risks and there is safe and effective treatment available for the disease. Prophylaxis can be provided on a voluntary basis (informed decision made by participant) or made a prerequisite for participation in HLC.
7. Participants should be monitored for signs of active disease throughout the course of their participation in the research. When possible, and feasible, researchers should have a mechanism established before starting the research for assuring treatment (if available) for any vector-borne disease that might be present in the study area.
8. Once participation is completed, the participant should be screened again for active disease immediately after and a set period of time following the study, and referred for treatment following national guidelines as necessary. The follow-up period is disease dependent. For malaria, 4 weeks after study exposure would be typical, for dengue 2 weeks; however, for filariasis, this period could be up to 1 year (3–12 months, depending on nematode species) before detecting a blood-patent infection. A serological diagnosis might be possible during prepatency, in which case treatment could be attempted.

**FIG. 5.**
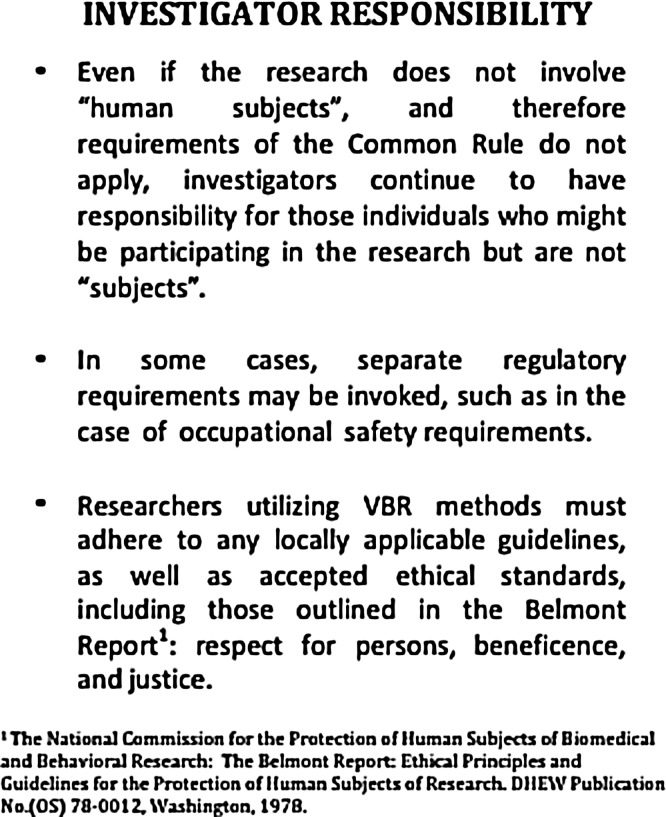
Investigator responsibility.

## Supplementary Material

Supplemental data

Supplemental data

## References

[B1] Alonso PL, Brown G, Arevalo-Herrera M, Binka F, et al. A research agenda to underpin malaria eradication. PLoS Med 2010; 8:e1000406 21311579 10.1371/journal.pmed.1000406 PMC3026687

[B2] American Committee on Arthropod-borne Viruses. The Subcommittee on Arbovirus Laboratory Safety: Laboratory safety for arboviruses and certain other viruses of vertebrates. Am J Trop Med Hyg 1980; 29:1359–1381 6778230 10.4269/ajtmh.1980.29.1359

[B3] Andrade-Narvaez F, Canto-Lara SB, Del Rosario Garcia-Miss M Leishmaniasis entomological field studies: Ethical issues. Dev World Bioeth 2009; 9:157–160 20021495 10.1111/j.1471-8847.2008.00245.x

[B4] Aultman KS, Walker ED, Gifford F, Severson DW, et al. Research ethics. Managing risks of arthropod vector research. Science 2000; 288:2321–2322 10917830 10.1126/science.288.5475.2321

[B5] Bailey NTJ The Biomathematics of Malaria. London: Oxford University Press, 1982

[B6] Beier JC Vector incrimination and entomological inoculation rates. Methods Mol Med 2002; 72:3–11 12125127 10.1385/1-59259-271-6:01

[B7] Belsky L, Richardson HS Medical researchers ancillary clinical care responsibilities. BMJ 2004; 328:1494–1496 15205296 10.1136/bmj.328.7454.1494 PMC428526

[B8] Bernier UR, Kline DL, Allan SA, Barnard DR Laboratory comparison of *Aedes aegypti* attraction to human odors and to synthetic human odor compounds and blends. J Am Mosq Control Assoc 2007; 23:288–293 17939508 10.2987/8756-971X(2007)23[288:LCOAAA]2.0.CO;2

[B9] Bhattarai A, Ali AS, Kachur SP, Mårtensson A, et al. Impact of artemisinin-based combination therapy and insecticide-treated nets on malaria burden in Zanzibar. PLoS Med 2007; 4:e309 17988171 10.1371/journal.pmed.0040309 PMC2062481

[B10] Chizema-Kawesha E, Miller JM, Steketee RW, Mukonka VM, et al. Scaling up malaria control in Zambia: Progress and impact 2005–2008. Am J Trop Med Hyg 2010; 83:480–488 20810807 10.4269/ajtmh.2010.10-0035 PMC2929038

[B11] Council for International Organizations of Medical Sciences (CIOMS). International Ethical Guidelines for Biomedical Research Involving ‘human subjects’. Biomedical Research 2002 Available at www.cioms.ch/14983848

[B12] Department of Health Education and Welfare (DHEW) National Commission for the Protection of ‘human subjects’ of Biomedical and Behavioral Research. The Belmont Report: Ethical Principles and Guidelines for the Protection of ‘human subjects’ of Research; Washington, DC: Publication no. (OS) 78-0012, 1978 25951677

[B13] Dietz K, Molineaux L, Thomas A A malaria model tested in the African savannah. Bull World Health Organ 1974; 50:347–357 4613512 PMC2481186

[B14] Dye C Vectorial capacity: Must we measure all its components. Parasitol Today 1996; 2:203–209 15462840 10.1016/0169-4758(86)90082-7

[B15] Eckhoff PA A malaria transmission-directed model of mosquito life cycle and ecology. Malaria J 2011; 10 17;10:303. doi: 10.1186/1475-2875-10-303 PMC3224385 21999664

[B16] Enayati A, Hemingway J Malaria management: Past, present, and future. Annu Rev Entomol 2010; 55:569–591 19754246 10.1146/annurev-ento-112408-085423

[B17] Fegan GW, Noor AM, Akhwale WS, Cousens S, et al. Effect of expanded insecticide-treated bednet coverage on child survival in rural Kenya: A longitudinal study. Lancet 2007; 370:1035–1039 17889242 10.1016/S0140-6736(07)61477-9 PMC2117339

[B18] Ferguson HM, Ng'habi KR, Walder T, Kadungula D, et al. Establishment of a large semi-field system for experimental study of African malaria vector ecology and control in Tanzania. Malaria J 2008; 7:158 10.1186/1475-2875-7-158 PMC2543042 18715508

[B19] Freier JE Estimation of vectorial capacity: Vector abundance in relation to man. Bull Soc Vect Ecol 1989; 14:41–46

[B20] Garrett-Jones C Prognosis for interruption of malaria transmission through assessment of the mosquito's vectorial capacity. Nature 1964; 204:1173–1175 14268587 10.1038/2041173a0

[B21] Garrett-Jones C The human blood index of malarial vectors in relationship to epidemiological assessment. Bull World Health Organ 1974; 30:241–261 14153413 PMC2554803

[B22] Garrett-Jones C, Shidrawi GR Malaria vectorial capacity of a population of *Anopheles gambiae*—an exercise in epidemiological entomology. Bull World Health Organ 1969; 40:531–545 5306719 PMC2556109

[B23] Gimnig JE, Walker ED, Otieno P, Kosgei J, et al. Incidence of malaria among mosquito collectors conducting human landing catches in Western Kenya. Am J Trop Med Hyg 2013; 88:301–308 23249685 10.4269/ajtmh.2012.12-0209 PMC3583321

[B24] Githeko AK, Mbogo CN, Curtis CF, Lines J, et al. Entomological monitoring of large-scale vector-control interventions. Parasitol Today 1996; 12:127–128

[B25] Grieco JP, Achee NL, Chareonviriyaphap T, Suwonkerd W, et al. A new classification system for the actions of IRS chemicals traditionally used for malaria control. PLoS One 2007; 8:e716 17684562 10.1371/journal.pone.0000716 PMC1934935

[B26] Gubler DJ Resurgent vector-borne diseases as a global health problem. Emerg Inf Dis 1998; 4:442–450 10.3201/eid0403.980326 PMC2640300 9716967

[B27] Herwaldt BL Laboratory-acquired parasitic infections from accidental exposures. Clin Microbiol Rev 2001; 14:659–688 11585780 10.1128/CMR.14.3.659-688.2001 PMC88999

[B28] Hooper CR Ancillary care duties: The demands of justice. J Med Ethics 2010; 36:708–711 20675740 10.1136/jme.2010.035758

[B29] Husbands RC Light traps and the significance of collection data. Bull Soc Vec Ecol 1976; 3:17–26

[B30] Institute of Medicine (US) Forum on Microbial Threats. Vector-Borne Diseases: Understanding the Environmental, Human Health, and Ecological Connections, Workshop Summary. Summary and Assessment. Washington (DC): National Academies Press, 2008 Available at www.ncbi.nlm.nih.gov/books/NBK52939/21452451

[B31] James S, Takken W, Collins FH, Gottlieb M Needs for monitoring mosquito transmission of malaria in a pre-elimination world. Am J Trop Med Hyg 2014; 90:6–10 24277786 10.4269/ajtmh.13-0175 PMC3886429

[B32] Kamau E, Alemayehu S, Feghali KC, Komisar J, et al. Measurement of parasitological data by quantitative real-time PCR from controlled human malaria infection trials at the Walter Reed Army Institute of Research. Malar J 2014; 13:288 25066459 10.1186/1475-2875-13-288 PMC4128310

[B33] Kilama M, Smith DL, Hutchinson R, Kigoz R, et al. Estimating the annual entomological inoculation rate for *Plasmodium falciparum* transmitted by *Anopheles gambiae* s.l. using three sampling methods in three sites in Uganda. Malaria J 2014; 13:111 10.1186/1475-2875-13-111 PMC4001112 24656206

[B34] Kilama WL Health research ethics in malaria vector trials in Africa. Mal J 2010; 9(Suppl 3):S3 10.1186/1475-2875-9-S3-S3 PMC3002141 21144083

[B35] Klein TA, Lima JB, Tada MS, Miller R Comparative susceptibility of anopheline mosquitoes in rondonia, Brazil to infection by *Plasmodium vivax*. Am J Trop Med Hyg 1991; 45:463–470 1951854 10.4269/ajtmh.1991.45.463

[B36] Lacroix R, Delatte H, Hue T, Reiter P Dispersal and survival of male and female *Aedes albopictus* (Diptera: Culicidae) on Réunion Island. J Med Entomol 2009; 46:1117–1124 19769043 10.1603/033.046.0519

[B37] Leitner WW, Costero-Saint Denis A, Wali T Immunological consequences of arthropod vector-derived salivary factors. Eur J Immunol 2011; 41:3396–3400 22125007 10.1002/eji.201190075

[B38] Lima JBP, Rosa-Freitas MG, Rodovalho CM, Santos F, et al. Is there an efficient trap or collection method for sampling *Anopheles darlingi* and other malaria vectors that can describe the essential parameters affecting transmission dynamics as effectively as human landing catches?—A Review. Mem Inst Oswaldo Cruz 2014; 109:685–705 25185008 10.1590/0074-0276140134 PMC4156462

[B39] Lindsay SW, Armstrong Schellenberg JR, Zeiler HA, Daly RJ, et al. Exposure of Gambian children to *Anopheles gambiae* malaria vectors in an irrigated rice production area. Med Vet Entomol 1995; 9:50–58 7696688 10.1111/j.1365-2915.1995.tb00116.x

[B40] Lyons AG The human dengue challenge experience at the Walter Reed Army Institute of Research. J Infect Dis 2014; 209(Suppl 2):S49–S55 24872396 10.1093/infdis/jiu174

[B41] Mabaso ML, Sharp B, Lengeler C Historical review of malaria control in southern African with emphasis on the use of indoor residual house-spraying. Trop Med Int Health 2004; 9:846–856 15303988 10.1111/j.1365-3156.2004.01263.x

[B42] Macdonald G The Epidemiology and Control of Malaria. London: Oxford University Press, 1957:121–149

[B43] McCabe ET, Barthel W.F, Gertler SI, Hall SA. Insect repellents III. *N*,*N*-diethylamides. J Org Chem 1954; 19:493–498

[B44] Mboera LE Sampling techniques for adult Afrotropical malaria vectors and their reliability in the estimation of entomological inoculation rate. Tanzanian Health Res Bull 2005; 7:117–124 10.4314/thrb.v7i3.14248 16941936

[B45] Murray C, Rosenfeld L, Lim S, Andrews K, et al. Global malaria mortality between 1980 and 2010: A systematic analysis. Lancet 2012; 379:413–431 22305225 10.1016/S0140-6736(12)60034-8

[B46] Najera JA A critical review of the field application of a mathematical model of malaria eradication. Bull Wld Hlth Organ 1974; 50:449–457 PMC2481134 4156197

[B47] Ng'habi KR, Mwasheshi D, Knols BG, Ferguson HM Establishment of a self-propagating population of the African malaria vector *Anopheles arabiensis* under semi-field conditions. Malar J 2010; 9:356 21143870 10.1186/1475-2875-9-356 PMC3017536

[B48] Okumu FO, Killeen GF, Ogoma S, Biswaro L, et al. Development and field evaluation of a synthetic mosquito lure that is more attractive than humans. PLoS One 2010; 5:e8951 20126628 10.1371/journal.pone.0008951 PMC2812511

[B49] Onari E, Grab B Indicators for forecasting of malaria epidemics. Bull World Health Organ 1980; 58:91–98 6966545 PMC2395888

[B50] Prasad RS Nutrition and reproduction in haematophagous arthorpods. Proc Indian Acad Sci (Anim Sci) 1987; 96:253–273

[B51] Pull JH, Grab B A simple epidemiological model for evaluating the malaria inoculation rate and the risk of infection in infants. Bull World Health Organ 1974; 51:507–516 4549501 PMC2366319

[B52] Roberts DR, Manguin S, Mouchet J DDT house spraying and re-emerging malaria. Lancet 2000; 356:330–332 11071203 10.1016/s0140-6736(00)02516-2

[B53] Sahu SS Comparative susceptibility of *Anopheles subpictus* from fresh and brackish water areas to *Plasmodium falciparum* infection. Acta Tropica 1998; 70:1–7 9707359 10.1016/s0001-706x(97)00140-x

[B54] Saul AJ, Graves PM, Kay BH A cyclical feeding model for pathogen transmission and its application to determine vectorial capacity from vector infection rates. J Appl Ecol. 1990; 90:123–133

[B55] Seder RA, Chang LJ, Enama ME, Zephir KL, et al. Protection against malaria by intravenous immunization with a nonreplicating sporozoite vaccine. Science 2013; 20;341:1359–1365 23929949 10.1126/science.1241800

[B56] Service MW A critical review of procedures for sampling populations of adult mosquitoes. Bull Entomol Res 1977; 67: 343–382

[B57] Sharp BL, Ridl FC, Govender D, Kuklinski J, et al. Malaria vector control by indoor residual insecticide spraying on the tropical island of Bioko, Equatorial Guinea. Malar J 2007; 6:52 17474975 10.1186/1475-2875-6-52 PMC1868751

[B58] Silver JB Mosquito Ecology: Field Sampling Methods, 3rd ed. Dordrecht: Springer Verlag, 2008

[B59] Smallegange RC, Knols BGJ, Takken W Effectiveness of synthetic versus natural human volatiles as attractants for *Anopheles gambiae* (Diptera: Culicidae) sensustricto. J Med Entomol 2010; 47:338–344 20496580 10.1603/me09015

[B60] Smith A Deterrent effect of insecticides on malaria vectors (rebuttal). Nature 1963; 200:861–862 10.1038/200860a0 14096060

[B61] Smith A, Webley DJ A verandah-trap hut for studying the house-frequenting habits of mosquitoes and for assessing insecticides. III. The effect of DDT on behavior and mortality. Bull Entomol Res 1969; 59:33–46 4390166 10.1017/s000748530000300x

[B62] Townson H, Nathan MB, Zaim M, Guillet P, et al. Exploiting the potential of vector control for disease prevention. Bull World Health Organ 2005; 83:942–947 16462987 PMC2626501

[B63] United States Environmental Protection Agency (EPA). Product Performance Test Guidelines OPPTS 810.3700 Insect Repellents For Human Skin and Outdoor Premises. 1999

[B64] United States Department of Health and Human Services. Code of Federal Regulations. Protection of Human Subjects, 45 C.F.R. Sect. 46 (2009). Printing Office, 2005

[B65] Vaughan JA, Bell JA, Turell MJ, Chadee DD Passage of ingested *Mansonella ozzardi* (Spirurida: Onchocercidae) microfilariae through the midgut of *Aedes aegypti* (Diptera: Culicidae). J Med Entomol 2007; 44:111–116 17294928 10.1603/0022-2585(2007)44[111:poimos]2.0.co;2

[B66] Wallace DI, Southworth BS, Shi X, Chipman JW, et al. A comparison of five malaria transmission models: Benchmark tests and implications for disease control. Malaria J 2014; 13:268 10.1186/1475-2875-13-268 PMC4105118 25011942

[B67] Weaver SC, Reisen WK Present and future arboviral threats. Antiviral Res 2010; 85:328–345 19857523 10.1016/j.antiviral.2009.10.008 PMC2815176

[B68] World Health Organization. Manual on Practical Entomology in Malaria. Part II. Methods and Techniques. Geneva: World Health Organization, 1975

[B69] World Health Organization. Guidelines for Testing Mosquito Adulticides for Indoor Residual Spraying and Treatment of Mosquito Nets. WHO/CDS/NTD/WHOPES/GCDPP/2006.3 Geneva: World Health Organization, 2006

[B70] World Health Organization. International Ethical Guidelines for Epidemiological Studies. Biomedical Research 1-113. Geneva; World Health Organization, 2008

[B71] World Health Organization. Guidelines for Efficacy Testing of Household Insecticide Products—Mosquito Coils, Vaporizer Mats, Liquid Vaporizers, Ambient Emanators and Aerosols. WHO/CDS/NTD/WHOPES/2009.3 Geneva: World Health Organization, 2009a

[B72] World Health Organization. Guidelines for Efficacy Testing of Mosquito Repellents for Human Skin. WHO/CDS/NTD/WHOPES/2009.4 Geneva: World Health Organization, 2009b

[B73] World Health Organization. Innovative Vector Control Interventions—2009 Annual Report. Geneva: World Health Organization, 2010

[B74] World Health Organization. World Malaria Report. Available at www.who.int/malaria/publications/world_malaria_report_2013/report/en/Geneva: World Health Organization, 2013a

[B75] World Health Organization. Guidelines for Laboratory and Field Testing of Long-Lasting Insecticidal Mosquito Nets. WHO/HTM/NTD/WHOPES/2013. Geneva: World Health Organization, 2013b

[B76] World Health Organization. Guidelines for Efficacy Testing of Spatial Repellents. WHO/HTM/NTD/WHOPES/2013.1 Geneva: World Health Organization, 2013c

[B77] Zulueta J, Cullen JR Deterrent effect of insecticides on malaria vectors. Nature 1963; 200:860–861 14096060 10.1038/200860a0

