# Supplementary material for: Considerations for the Use of Human Participants in Vector Biology Research: A Tool for Investigators and Regulators
Source: Vector Borne Zoonotic Dis. 2015 Feb 1;15(2):89–102. doi: 10.1089/vbz.2014.1628 (PMC4340630; doi:10.1089/vbz.2014.1628)
Supplement: Supplemental data [file Supp_Appendix1.pdf]

Appendix 1. Cosiderations for Vector Biology Research activities with human participation.

Vector collection: Collections can involve a manipulation of the participant's environment, however, the collection of vectors from a person or a person's environment does not in itself constitute information about the individual. Therefore, the collection procedure itself does not involve 'human subjects'.

Blood test: Venipuncture or finger-stick is described as a physical procedure by which data are gathered, and is therefore considered to be an intervention. The principal question is whether venipuncture is being performed for research purposes or only as a standard medical procedure to ensure participant's safety.

Ensuring safety: When testing is done to ensure the safety of the participant (i.e., to identify infection so it can be treated) or the community (i.e., to prevent vectors from becoming infected), and the results of the testing will not be used in pursuit of the research objectives, such a procedure is not considered to be for research purposes and it does not in itself involve 'human subjects'.

Assessing participant characteristics: Any testing to assess participant characteristics in pursuit of research objectives – whether those characteristics are to be assessed as outcomes or as co-variants – is considered to be for research purposes, and therefore the research would involve 'human subjects'.

Prophylaxis/Treatment: The provision of prophylaxis or treatment is considered an intervention. The principal question is whether it is provided for research purposes or only as a standard medical procedure to ensure participant's safety. Ensuring safety: When prophylaxis or treatment is provided to ensure the safety of the participant, it is not in itself considered to be for research purposes. Assessed as a co-variate: When the provision of prophylaxis or treatment is to be assessed as a co-variate in pursuit of research objectives, it is considered to be for research purposes, and therefore would involve 'human subjects'.

Data collection:

Environment: Data recorded about the environment in which the HLC take place is not information about the individual. On its own, this would not involve 'human subjects'.

Attractant: Any information about the participant, including physical characteristics, health status, knowledge, attitudes, practices, opinions, preferences, etc. that is to be assessed in pursuit of research objectives is considered to be for research purposes. Collection of such information, whether through observation or through interaction is considered research involving 'human subjects'.

Administrative data: Collection of personal identifiers (and occasionally limited demographic data) solely for administrative purposes (e.g., for provision of payment) is not considered to be information collected for research.

Activity	Procedure	Decision Points				Outcome	
		**Is data collected about the individual for research purposes?**	**Will data about the individual be collected through intervention?**	**Will data about the individual be collected through interaction?**	**Will identifiable private information be used for research purposes?**	**Human Subjects?**	**Risk Assessment:**<br>**Does increased risk exist?**
Human Landing Catches:<br>Natural environment (forest, inside or outdoors of local house etc.)	Vector Attraction and Collection	**Yes:** If data is collected *about the individual* for purposes of research.<br><br><u>EXAMPLE:</u> Measuring the rate of pathogen infection in collectors vs. non-collectors.	**Yes:** If data *about the individual* are collected through physical procedures (e.g., finger stick).	→		**Yes:** Information is collected *about* the individual for purposes of research.<br><br>**Follow regulations governing the protection of human research subjects (45 CFR 46, Subpart A).**	**Yes:** In disease endemic areas.<br><br>**Risk Mitigation:** Yes<br>**(See suggestions)**
			**No:** If no data about the individual are collected through physical procedures.	**Yes:** If data about the individual will be collected through interaction (e.g. survey).	→		
				**No:** If no data about the individual are collected through interaction.	**Yes:** If data from other sources (e.g., medical records) that are both identifiable and private are used for research purposes.<br><br>Or if observations about the individual's behavior in a private setting are recorded in an identifiable manner.		
		**No:** If data is not collected *about* the individual for purposes of research.<br><br><u>EXAMPLE:</u> Measuring vector species composition, or indoor/outdoor biting patterns.	*Because no information about the individual is being collected, this would not be considered to involve human subjects, even though there may be interaction or intervention for the purposes of the research.*<br><br>→			**No**: Information is not collected *about* the individual for purposes of research. Additional protections related to occupational exposure (i.e., potential bites) and health should be considered.	

<table>
<tr><th></th><th></th><th colspan="4">Decision Points</th><th colspan="2">Outcome</th></tr>
<tr><th>Activity</th><th>Procedure</th><th>Is data collected about the individual for research purposes?</th><th>Will data about the individual be collected through intervention?</th><th>Will data about the individual be collected through interaction?</th><th>Will identifiable private information be used for research purposes?</th><th>Human Subjects?</th><th>Risk Assessment:
Does increased risk exist?</th></tr>
<tr><td rowspan="4">Human Landing Catches:
Experimental environment
(newly constructed hut; biosphere (i.e., screened enclosure))</td><td rowspan="4">Vector Attraction
and
Collection</td><td rowspan="3">Yes: If data is collected about the individual for purposes of research.

<u>EXAMPLE:</u> Measuring the effect of gender, age, underlying illness etc., on density of mosquitoes entering a hut.</td><td>Yes: If there is collection of information about the individual through physical procedures (e.g., venipuncture);

Or if observations about the individual's behavior within the manipulated environment are recorded.</td><td colspan="2">→</td><td rowspan="3">Yes: Information is collected about the individual for purposes of research.

Follow regulations governing the protection of human research subjects (45 CFR 46, Subpart A).</td><td rowspan="4">Yes: When huts are outside screened enclosures in disease endemic areas.

No: When using biosphere and uninfected vectors.

Risk Mitigation: Yes under both scenarios.
(See suggestions)</td></tr>
<tr><td rowspan="2">No: If no information about the individual are collected through such procedures.</td><td>Yes: If information about the individual is collected through interaction (e.g., a survey).</td><td>→</td></tr>
<tr><td>No: If no data about the individual are collected through interaction.</td><td>Yes: If data from other sources (e.g., medical records) that are both identifiable and private are used for research purposes.

Or if observations about the individual's behavior in a private setting are recorded in an identifiable manner.</td></tr>
<tr><td>No: If data is not collected about the individual for purposes of research.

<u>EXAMPLE:</u> Measuring density, time of mosquito entry, exit to compare between control and intervention.</td><td colspan="3">Although this type of study necessarily involves a manipulation of the environment, it would not involve human subjects unless information about the individual was obtained for research purposes.

→</td><td>No: Information is not collected about the individual for purposes of research. Additional protections related to occupational exposure (i.e., potential bites) and health should be considered.</td></tr>
</table>

<table>
<tr><th rowspan="2">Activity</th><th rowspan="2">Procedure</th><th colspan="4">Decision Points</th><th colspan="2">Outcome</th></tr>
<tr><th>Is data collected about the individual for research purposes?</th><th>Will data about the individual be collected through intervention?</th><th>Will data about the individual be collected through interaction?</th><th>Will identifiable private information be used for research purposes?</th><th>Human Subjects?</th><th>Risk Assessment:
Does increased risk exist?</th></tr>
<tr><td rowspan="4">Trapping:
Natural environment
(CDC light traps, oviposition traps, or similar, inside or outdoors of local house)</td><td rowspan="4">Vector Attraction and Collection</td><td rowspan="3">Yes: If data is collected about individuals for purposes of research.

<u>EXAMPLE:</u> Measuring effect of vector densities in relation to characteristics of specific house occupants.</td><td>Yes: If observations about individuals' behavior related to trap are recorded.

Or if data are collected about individuals through physical procedures.</td><td colspan="2">→</td><td rowspan="3">Yes: Information is collected about the individual for purposes of research.

Follow regulations governing the protection of human research subjects (45 CFR 46, Subpart A).</td><td rowspan="4">No</td></tr>
<tr><td rowspan="2">No: If such information is not recorded or collected.</td><td>Yes: If information about an individual is obtained through survey/interview/etc. with that individual.</td><td>→</td></tr>
<tr><td>No: If no such information is obtained.</td><td>Yes: If identifiable private information about individuals is collected (e.g., through proxy interviews or through existing records).

Or if observations about the individual's behavior in a private setting are recorded in an identifiable manner.</td></tr>
<tr><td>No: If data is not collected about individuals for purposes of research.

<u>EXAMPLE:</u> Measuring vector species density, composition, or general distribution patterns in a given location.</td><td colspan="3">Although this type of activity usually involves intervention, the question is whether the intervention leads to information about the individual receiving the intervention. Observations directly related to the intervention should be considered information collected through intervention.

Surveys involve human subjects if the respondent is asked to report information about him or herself, or if the respondent is asked to provide identifiable private information about others.

Observations not directly related to the intervention involve human subjects only if they are about behavior in a private setting and are identifiable.

→</td><td>No: Information is not collected about the individual for purposes of research.</td></tr>
</table>

<table>
<tr><th rowspan="2">Activity</th><th rowspan="2">Procedure</th><th colspan="4">Decision Points</th><th colspan="2">Outcome</th></tr>
<tr><th>Is data collected about the individual for research purposes?</th><th>Will data about the individual be collected through intervention?</th><th>Will data about the individual be collected through interaction?</th><th>Will identifiable private information be used for research purposes?</th><th>Human Subjects?</th><th>Risk Assessment:
Does increased risk exist?</th></tr>
<tr><td rowspan="4">Use of biospheres (i.e., screened enclosure) where humans sleep in order to capture and/or provide blood meals to release populations of <u>non-infective</u> mosquitoes.</td><td rowspan="4">Vector Release, Collection and/or Feeding</td><td rowspan="3">Yes: If data is collected about the individual for purposes of research.

<u>EXAMPLE:</u> Measuring the effect of gender, age, underlying illness etc., on density of mosquitoes entering a hut or taking a blood meal.</td><td>Yes: If observations about the individual's behavior related to a manipulated environment are recorded.

Or if data are collected about the individual through physical procedures.</td><td colspan="2">→</td><td rowspan="3">Yes: Information is collected about the individual for purposes of research.

Follow regulations governing the protection of human research subjects (45 CFR 46, Subpart A).</td><td rowspan="4">No: When using biosphere and uninfected vectors.

Risk Mitigation: Yes
(See suggestions)</td></tr>
<tr><td rowspan="2">No: If no such information is collected.</td><td>Yes: If the individual is asked to provide information about him/herself through survey/interview/etc.</td><td>→</td></tr>
<tr><td>No: If no such information is collected.</td><td>Yes: If data from other sources (e.g., medical records) that are both identifiable and private are used for research purposes.

Or if observations about the individual's behavior in a private setting are recorded in an identifiable manner.</td></tr>
<tr><td>No: If data is not collected about the individual for purposes of research.

<u>EXAMPLE:</u> Measuring time of mosquito entry or successful blood meal acquisition to compare between control and intervention.</td><td colspan="3">Although this type of activity usually involves intervention, the question is whether the intervention leads to information about the individual. Observations directly related to the intervention should be considered information collected through intervention.

Surveys involve human subjects if the respondent is asked to report information about him or herself.

Observations not directly related to the intervention involve human subjects only if they are about behavior in a private setting and are identifiable.

→</td><td>No: Information is not collected about the individual for purposes of research. Additional protections related to occupational exposure (bites) and health should be considered.</td></tr>
</table>

<table>
<thead>
<tr><th rowspan="2">Activity</th><th rowspan="2">Procedure</th><th colspan="4">Decision Points</th><th colspan="2">Outcome</th></tr>
<tr><th>Is data collected about the individual for research purposes?</th><th>Will data about the individual be collected through intervention?</th><th>Will data about the individual be collected through interaction?</th><th>Will identifiable private information be used for research purposes?</th><th>Human Subjects?</th><th>Risk Assessment:
Does increased risk exist?</th></tr>
</thead>
<tbody>
<tr><td rowspan="4">Feeding uninfected vectors on humans infected with a pathogen of interest prior to clinical treatment.</td><td rowspan="4">Vector Feeding</td><td rowspan="3">Yes: If data is collected about the individual for purposes of research.

<u>EXAMPLE:</u> Measuring the influence of blood type, partial acquired immunity, or the effect of HIV infection on development of the pathogen in the vector.</td><td>Yes: If information about the individual will be obtained through a physical procedure (e.g., venipuncture for diagnostics) performed for the purpose of a specified research objective.

Or if observations about an individual's behavior in a manipulated environment are recorded.</td><td colspan="2">→</td><td rowspan="3">Yes: Information is collected about the individual for purposes of research.

Follow regulations governing the protection of human research subjects (45 CFR 46, Subpart A).</td><td rowspan="3">No

Risk Mitigation: Yes
(See suggestions)</td></tr>
<tr><td rowspan="2">No: If no such information is obtained.</td><td>Yes: If information about the individual is obtained through survey/interview/etc.</td><td>→</td></tr>
<tr><td>No: If no such information is obtained.</td><td>Yes: If data from other sources (e.g., medical records) that are both identifiable and private are used for research purposes.

Or if observations about the individual's behavior in a private setting are recorded in an identifiable manner.</td></tr>
<tr><td>No: If data is not collected about the individual for purposes of research.

<u>EXAMPLE:</u> Measuring infection rates among various vector species to determine vector competency.</td><td colspan="3">Although this type of activity usually involves the selection of individuals based on one or more specific characteristics (e.g., gender, age, parasitemia, etc.), the question is whether that information is being used in pursuit of an explicit research objective.

→</td><td>No: Information is not collected about the individual for purposes of research.</td><td>No

Risk Mitigation: Yes
(See suggestions)</td></tr>
</tbody>
</table>
