# Supplementary material for: Considerations for the Use of Human Participants in Vector Biology Research: A Tool for Investigators and Regulators
Source: Vector Borne Zoonotic Dis. 2015 Feb 1;15(2):89–102. doi: 10.1089/vbz.2014.1628 (PMC4340630; doi:10.1089/vbz.2014.1628)
Supplement: Supplemental data [file Supp_Appendix2.docx]

##### Appendix 2. Applications and Impact of Using Human-Landing Catch (HLC) in Vector Biology Research

***It is important to note that the risk assessment for each example is limited to those risks related to the vector; the examples provided have other risks, but typically reviewers are familiar with those risks and how to assess them in the context of the overall benefits of vector biology research (VBR).***

- 1. ***Case Study 1 (Phase I; Cage-trials and Permethrin Insecticide Treated Materials (ITM)***
     - Background: In August, 2003, a military operation to secure an airstrip in Monrovia, Liberia failed because 80 of the 150 soldiers, mostly Marines, apparently were infected with malaria parasites.1 The total cost for medical treatment of these Marines was $1,483,120. Proper factory treatment of their uniforms with permethrin would have cost $11,600, a savings of over $1.4 million for this single, small-scale operation. The USMC asked USDA in 2005 to devise a test to ensure that their treated military uniforms were: (1) as insect-proof as possible; (2) contained an appropriate level of permethrin to provide a high level of bite protection from mosquitoes and; (3) that this high level of protection lasted throughout the expected uniform lifetime (~50 wash cycles).
     - Methodologies (vector measures): From 2004–2005, USDA examined the garments using mosquito “knockdown” (KD) analysis of the fabric, a common practice at the time that includes measuring the number of mosquitoes that were effectively unable to fly after making contact with treated material for a set exposure time (*i.e*., 30 m to 1 h). It was discovered that KD analysis was too imprecise and not an accurate predictor of the bite protection afforded by treated military uniforms. The KD test is designed to determine insecticidal activity as a toxicant and the ability of the test compound to ultimately kill the target vector. The application of permethrin on the uniform is, however, intended to perform primarily as a repellent that prevents vector biting, thus reducing the risk of transmission. The USDA findings showed that the best assessment of bite protection performance was through use of human volunteers wearing permethrin-treated fabric sleeves inside cage tests as compared with untreated controls in preventing biting. The methods used were those described in the *EPA Product Performance Guidelines*2 in which biting pressure is measured before treatment and every hour during the test. The test subject’s forearm, wrist to elbow, is used as the test area. Pathogen-free, colony-reared mosquitoes are allowed to bite or probe for 5 minutes or until the recommended number of bites occurs (5 bites in 5 min for mosquitoes) with continuous exposure for timed duration of test.
     - Risk assessment: No risk of arthropod-borne disease was possible as only pathogen-free colony-reared mosquito test populations were used. Possible adverse side effects may include minor, transient and localized allergic reaction to bites. Subjects with history of allergic reactions to mosquito bites were excluded.
     - Risk mitigation:
       - Use of colony-reared mosquitoes, so the risk of acquiring a vector-borne disease is eliminated.
       - Use of human volunteers. The Federal Insecticide, Fungicide, and Rodenticide Act (FIFRA) Section 12(a)(2)(P) defines it as unlawful ‘‘for any person to use any pesticide in tests on human beings unless such human beings are fully informed of the nature and purposes of the test and of any physical and mental health consequences which are reasonably foreseeable therefore, and freely volunteer to participate in the test.’’ 40 CFR 26.116 outlines the elements of informed consent to participate in the test (study).
       - Those areas above and below the forearm are covered with a material that prevents the mosquito proboscis from penetration. Dark-colored material to be avoided. Hands may be covered with latex gloves.
     - Outcome/advances to VBR:
       - A protocol has been devised to conduct similar ITM tests and this protocol has been used to qualify companies to provide permethrin factory-treat clothing Advanced Combat Uniform (ACUs) for the USMC.
       - Perhaps the most significant endorsement of this test approach is from the United States Environmental Protection Agency (US EPA) in their latest data call for permethrin-treated clothing that recommended the USDA protocol to registrants as the appropriate manner in which to evaluate repellent-treated clothing.

1. Whitman TJ, Coyne PE, Magill AJ, Blazes DL, et al. An outbreak of *Plasmodium falciparum* malaria in U.S. marines deployed to Liberia. Am J Trop Med Hyg 2010; 83:258[[[en dash]]]265.

2. United States Environmental Protection Agency (EPA). Product Performance Test Guidelines OPPTS 810.3700 Insect Repellents for Human Skin and Outdoor Premises. 1999.

1. ***Case Study 2 (Phase II; Natural Mosquito Populations): Experimental Huts and Indoor Residual Spray, Chemical Mode of Action1/ Insecticide-treated Bednet Efficacy2***
   - Purpose: To measure the efficacy of chemical products designed to reduce vector entry into treated structures and/or biting success of mosquitoes once indoors. Utilize experimental hut assays in a malaria-endemic area.
   - Methodologies (vector measures):
     - Adult volunteers enter huts at dusk and sleep under bed nets (for insecticide-treated net [ITN] evaluations) or perform interception trap collections (for ITN and/or indoor residual spray [IRS] evaluations) until dawn.
     - Mosquitoes are collected either once the next morning or throughout the night on an hourly basis: Dead mosquitoes are aspirated from the floor of the hut as well as from the exit traps and inside ITN (when used); resting mosquitoes are collected using aspirators from inside the ITN (when used) and from the walls and roof of the experimental hut and exit traps; entering/exiting mosquitoes are collected from window, door, or eave traps as appropriate.
     - Mosquitoes are scored by location and time as dead or alive and as blood “fed” or “unfed.” Live mosquitoes are placed in small holding cups and provided sugar solution as sustenance for 24 h to assess delayed mortality.
   - Risk assessment: Volunteers may be exposed to naturally infected vector populations; however, in an endemic setting, exposure to infected mosquitoes is an ordinary risk of everyday life. As designed, the study does not introduce additional risk of exposure than participants would experience if they did not participate.
   - Risk mitigation:
     - ITN evaluations: Volunteers sleep under an ITN, reducing exposure to infected mosquitoes. In addition, resting mosquitoes that find their way inside the net are collected, further reducing feeding opportunities.
     - IRS evaluations: Entering mosquitoes are collected via window traps, and resting mosquitoes that are able to enter are collected throughout the night, thereby reducing the number of mosquitoes inside the dwelling.
     - Effective chemoprophylaxis provided, if available and where appropriate, and volunteers medically supervised throughout and immediately following study period.
   - Outcome/advances to VBR: Guidance of efficacy testing for improvement of existing tools and development of future chemical products.
2. Grieco JP, Achee NL, Chareonviriyaphap T, Suwonkerd W, et al. A new classification system for the actions of IRS chemicals traditionally used for malaria control. PLoS One 2007; 8;2:e716..
3. N'Guessan R, Darriet F, Doannio JMC, Chandre F, et al. Olyset Net efficacy against pyrethroid-resistant *Anopheles gambiae* and *Culex quinquefasciatus* after 3 years' field use in Côte d'Ivoire. Med Vet Entomol 2001; 15:97[[[en dash]]]104.
4. ***Case Study 3 (Phase III; Natural Mosquito Populations): The Garki Project1***
   - Purpose: (1) To provide evidence-based administrative and technical guidelines for the planning, organization, and evaluation of malaria control campaigns; (2) to provide a detailed understanding of malaria epidemiology in the African (northern Nigeria) savanna environment through a multidisciplinary, longitudinal epidemiological study; and (3) to design more realistic mathematical models of the dynamics of malaria transmission using field-collected data.
   - Methodology (vector measures): The methods used are those described in the *Manual on Practical Entomology in Malaria.2* This includes: (1) All-night (12 h) human bait collections (12 h); (2) window exit trap collections fixed to houses; (3) pyrethrum spray collections inside homes; and (4) outdoor adult resting collections.
   - Risk assessment: [[[em dash]]]unknown before study (an actual component outcome of the study)
   - Risk mitigation: [[[em dash]]]unknown
   - Outcome/advances to VBR: “Construction of a mathematical model of malaria transmission that links entomological and parasitological variables, in particular vectorial capacity [the risk of transmission] and the prevalence of *Plasmodium falciparum* [in the human population], and of calculating the expected parasitological effect with changes in the entomological situation [parameters] [biting density, longevity, man-biting habit], natural or man-made.”1

1. Molineaux L, Gramiccia G. Chapter 10: The mathematical model of transmission] [A major field study from 1969 to 1976]. In: *The Garki Project: Research on the Epidemiology and Control of Malaria in the Sudan Savanna of West Africa*. Geneva: World Health Organization, 1980.
